# Artificial intelligence in Saudi otolaryngology research: a scoping review of the current landscape, gaps, and future directions

**DOI:** 10.3389/fsurg.2026.1983298

**Published:** 2026-10-21

**Authors:** Turki Aldrees, Abdullah Alabdulqader, Mohammed Al-Bar, Feras Alkholaiwi

**Affiliations:** 1 Department of Otolaryngology, Head and Neck Surgery, College of Medicine, Prince Sattam Bin Abdulaziz University, Alkharj, Saudi Arabia; 2 Department of Otolaryngology, Head and Neck Surgery, College of Medicine, Imam Mohammad Ibn Saud Islamic University, Riyadh, Saudi Arabia; 3 Department of Otolaryngology, Head and Neck Surgery, College of Medicine, Imam Abdulrahman Bin Faisal University, Dammam, Saudi Arabia

**Keywords:** artificial intelligence, machine learning, otolaryngology, Saudi Arabia, scoping review

## Abstract

**Background:**

Artificial intelligence (AI) is increasingly used in otolaryngology, but the extent and focus of AI research in Saudi Arabia remain unclear. This scoping review mapped the available evidence and identified key research themes and knowledge gaps.

**Methods:**

A scoping review was conducted following the PRISMA-ScR guidelines. PubMed/MEDLINE, Scopus, Web of Science, Google Scholar, and the Saudi Medical Journal archive were searched for studies published between January 2019 and April 2026. Eligible studies investigated AI applications or clinician perspectives within Saudi otolaryngology. The evidence was mapped across major otolaryngology domains, including cochlear implantation and hearing, rhinology and skull base surgery, facial plastic surgery, laryngology and voice, head and neck oncology, surgical and other ENT-related interventions for obstructive sleep apnoea, AI education and implementation, and AI-assisted patient education.

**Results:**

The searches identified 2,925 database records, four records from other sources and seven records from an updated search and expert suggestions; thirteen studies met the inclusion criteria. The evidence covered AI awareness and education, large language model (LLM)-based patient education, cochlear implantation, rhinology and skull base surgery, facial plastic surgery, laryngology and voice, and head and neck oncology; no eligible study addressed surgical or other ENT-related interventions for obstructive sleep apnoea. Clinicians were generally positive about AI, while clinical studies showed promising performance of AI models. LLM chatbots generally gave accurate postoperative advice, although, when validated tools were used, their responses were harder to read and less actionable than patient-education standards recommend. However, limited AI education, small or non-Saudi datasets, limited external validation, and implementation barriers remained important challenges.

**Conclusion:**

AI research in Saudi otolaryngology remains limited but is expanding across several clinical domains. Future research should prioritise Saudi-specific datasets, multicentre clinical validation, structured AI education, rigorous bilingual (Arabic and English) evaluation of LLM-generated patient information, and safe integration of AI into clinical practice in line with Saudi Vision 2030.

## Introduction

Artificial intelligence (AI), which includes machine learning (ML) and deep learning (DL), allows computers to learn patterns from complex clinical data and apply them to diagnosis, prediction and decision support (1–3). Otolaryngology is well suited to these methods because everyday practice generates endoscopic, radiological, audiological, voice and histopathological data (4, 5). AI has now been applied across most ENT subspecialties, from otoscopic image interpretation and otitis media diagnosis to cochlear implant outcome prediction, voice pathology classification, sinonasal imaging, head and neck tumour detection and sleep apnoea detection (6–10). More recently, large language models (LLMs) such as ChatGPT have added a new dimension, as patients and clinicians increasingly turn to them for medical information.

Saudi Arabia has made AI a national priority. Vision 2030 and the National Strategy for Data and Artificial Intelligence (NSDAI) have driven investment in digital health infrastructure and in AI applications across radiology, oncology, emergency medicine and population health (11, 12). Most AI research, however, still comes from the United States, China and Europe (13), and it is not clear how far Saudi otolaryngology has kept pace. Local implementation also faces familiar obstacles, including fragmented data, limited interoperability, regulatory and ethical questions, algorithmic bias and uneven clinician familiarity with AI (14, 15).

Saudi research on AI in otolaryngology has grown in recent years, but it is scattered across clinician surveys, a small number of model-development studies and, more recently, evaluations of LLM chatbots, and no review has brought it together. As a result, it is unclear which subspecialties have been studied, what kinds of AI and data have been used, and where the main gaps lie. This scoping review therefore aimed to map AI research in Saudi otolaryngology, describe the clinical domains and methods studied, identify gaps in the evidence, and suggest priorities for future research and for responsible clinical use in line with Saudi Vision 2030.

## Methods

### Study design

This scoping review followed the five-stage framework described by Arksey and O'Malley (16), with the refinements proposed by Levac et al. (17): identifying the research question; identifying relevant studies; selecting studies; charting the data; and collating, summarising and reporting the results. Reporting follows the Preferred Reporting Items for Systematic Reviews and Meta-Analyses extension for Scoping Reviews (PRISMA-ScR). The review was guided by three questions: (1) What AI research has been conducted in Saudi otolaryngology, and in which subspecialties? (2) What AI methods, data sources and study designs have been used? (3) What gaps exist, and what should future research and clinical implementation prioritise? We examined AI research across the major otolaryngology subspecialties (cochlear implantation and hearing, rhinology and skull base surgery, facial plastic surgery, laryngology and voice, head and neck oncology, and surgical and other ENT-related interventions for obstructive sleep apnoea), together with the cross-cutting themes of AI awareness and education, clinician perceptions and readiness, AI-assisted patient education, and barriers to adoption.

### Search strategy

Five sources were systematically searched: PubMed/MEDLINE, Scopus, Web of Science, Google Scholar, and the Saudi Medical Journal archive, covering studies published between January 2019 and April 2026. The search strategy was developed using controlled vocabulary, where applicable, and free-text terms combined with Boolean operators. The search strategy was designed to capture both established and emerging AI technologies and to ensure adequate representation of the major ENT subspecialties identified in the review objectives. An updated targeted search was then run over the same period. It added facial plastic and skull base terms (rhinoplasty, otoplasty, facelift, skull base, endonasal, cerebrospinal fluid leak), chatbot and model names (chatbot, Gemini, Bard, DeepSeek, Claude, Copilot, Llama, Perplexity, BERT, transformer), and a search of author affiliations. Reference lists of included studies were also checked, and studies suggested by content experts were screened against the same eligibility criteria.

Three major search concepts were combined:

1. **Artificial Intelligence:** (“artificial intelligence” OR “machine learning” OR “deep learning” OR “neural network*” OR “computer vision” OR “natural language processing” OR “ChatGPT” OR “large language model*” OR “generative AI”)
2. **Otolaryngology and subspecialty terms:** (otolaryngology OR otorhinolaryngology OR ENT OR otology OR neurotology OR audiology OR “cochlear implant*” OR “hearing loss” OR tinnitus OR vertigo OR rhinology OR rhinosinusitis OR sinusitis OR “chronic rhinosinusitis” OR “nasal polyp*” OR laryngology OR voice OR “voice disorder*” OR dysphonia OR dysphagia OR “vocal fold*” OR laryngoscop* OR “head and neck surgery” OR “head and neck cancer” OR oncology OR tumour OR tumor OR “oral cancer” OR “thyroid cancer” OR “nasopharyngeal carcinoma” OR “sleep apnea” OR “sleep apnoea” OR “obstructive sleep apnea” OR “obstructive sleep apnoea”)
3. **Geographical Context:** (“Saudi Arabia” OR Saudi OR “Kingdom of Saudi Arabia”). The search syntax was adapted for each database according to its indexing system and search interface. In PubMed, terms were searched in All Fields, which include author affiliations; in Scopus and Web of Science, searches were limited to titles, abstracts and keywords (TITLE-ABS-KEY and Topic fields, respectively).

### Study selection

All retrieved records were exported into Microsoft Excel, where duplicate citations were identified and removed. Study selection was conducted in two sequential stages. First, two reviewers (T.A. and A.A.) independently screened titles and abstracts against the predefined eligibility criteria (Table 1). Articles considered potentially relevant proceeded to full-text review. Second, the same two reviewers independently assessed the full texts for final eligibility. Disagreements at either stage were resolved by discussion with two senior reviewers (F.A. and M.A.). Reasons for exclusion during full-text assessment were documented. The overall screening process is presented using a PRISMA-ScR flow diagram.

**Table 1:** Eligibility criteria.

Inclusion criteria	Exclusion criteria
Studies investigating artificial intelligence (AI) applications in otolaryngology (ENT), head and neck surgery, audiology, rhinology, laryngology, obstructive sleep apnoea where the study addresses upper-airway assessment or a surgical or other ENT-related intervention (e.g., drug-induced sleep endoscopy, upper-airway surgery or hypoglossal nerve stimulation), or related ENT subspecialties, including facial plastic and skull base surgery performed by otolaryngology teams.	Studies unrelated to otolaryngology, head and neck disorders, or ENT clinical practice.
Studies conducted within a Saudi Arabian context, including those using Saudi patient data, conducted in Saudi healthcare institutions, involving Saudi healthcare professionals, or addressing AI implementation in Saudi otolaryngology.	Studies focusing exclusively on non-ENT medical specialties without relevance to otolaryngology, including sleep apnoea studies limited to event detection from physiological signals (e.g., electrocardiography or oximetry) without upper-airway assessment or a surgical or other ENT-related intervention.
Studies evaluating AI technologies such as artificial intelligence (AI), machine learning (ML), deep learning (DL), convolutional neural networks (CNNs), natural language processing (NLP), large language models (LLMs), computer vision, generative AI, or explainable AI.	Editorials, letters to the editor, opinion articles, commentaries, conference abstracts without full-text availability, study protocols, and dissertations.
Studies assessing AI for diagnosis, prediction, classification, prognosis, clinical decision support, image analysis, workflow optimisation, education, or clinician perceptions within otolaryngology.	Studies that did not evaluate AI applications or clinician perspectives related to AI in otolaryngology.
Original research studies, systematic reviews, and scoping reviews relevant to the research question.	Duplicate publications or studies with insufficient methodological information.
Articles published in the English language.	Articles published in languages other than English.
Articles published between January 2019 and April 2026 (based on the date of first online publication)	Articles published before January 2019 or after April 2026.
Full-text articles available for review.	Studies for which the full text was unavailable.

### Data extraction

Data extraction was performed using a standardised extraction form developed specifically for this review. Two reviewers (T.A. and A.A.) extracted the data independently, and any discrepancies were resolved by F.A. and M.A. The following information was extracted from each included study: author, year, ENT domain, country or context, study design, AI focus, participants or dataset, and main findings. Consistent with scoping review methodology, formal critical appraisal of the included studies was not performed.

### Data synthesis

A descriptive narrative synthesis was undertaken because of the substantial heterogeneity in study designs, AI methodologies, clinical applications, datasets, and outcome measures. The included studies were grouped, for the narrative synthesis, according to major otolaryngology subspecialties (cochlear implantation and hearing, rhinology and skull base surgery, facial plastic surgery, laryngology and voice, head and neck oncology, and surgical and other ENT-related interventions for obstructive sleep apnoea). Findings were additionally organised into an evidence map across four predefined thematic domains: AI awareness and educational preparedness; clinician perceptions and readiness for AI implementation; clinical AI development; and barriers to AI adoption. During data charting we added a fifth domain, AI-assisted patient education, because several of the included studies tested LLM chatbots as a source of information for patients. Quantitative pooling or meta-analysis was not performed because the review aimed to map the scope and characteristics of existing evidence rather than estimate pooled effect sizes.

## Results

### Search results

The electronic database search identified 2,925 records. PubMed yielded 2,912 records, while 7 records were identified through Scopus and 6 records through Web of Science. The much larger PubMed yield reflects its All Fields search, which also matches author affiliations and therefore retrieved many Saudi-affiliated papers unrelated to otolaryngology or AI; nearly all of these were excluded at title and abstract screening. In addition, four potentially relevant records were identified through supplementary searching of Google Scholar and the Saudi Medical Journal archive, together with reference checking. Following the removal of two duplicate records, 2,927 unique records underwent title and abstract screening. Of these, 2,919 records were excluded because they were unrelated to otolaryngology, did not involve artificial intelligence applications, lacked a Saudi Arabian context, or did not meet the predefined eligibility criteria. Eight full-text articles were subsequently assessed for eligibility. One article was excluded at the full-text stage because it was published after the predefined April 2026 search cutoff. Seven studies therefore met the inclusion criteria from the database search. One of them, a study detecting sleep apnoea from electrocardiographic signals, was later excluded after the eligibility criteria were refined to limit sleep apnoea studies to those addressing upper-airway assessment or a surgical or other ENT-related intervention. This left six studies from the database search. Seven further records were identified through the updated search and expert suggestions (citation searching). All were read in full, and all met the inclusion criteria. In total, thirteen studies were included in the final scoping review. The complete study selection process is illustrated in the PRISMA-ScR flow diagram (Figure 1).

**Figure 1:**
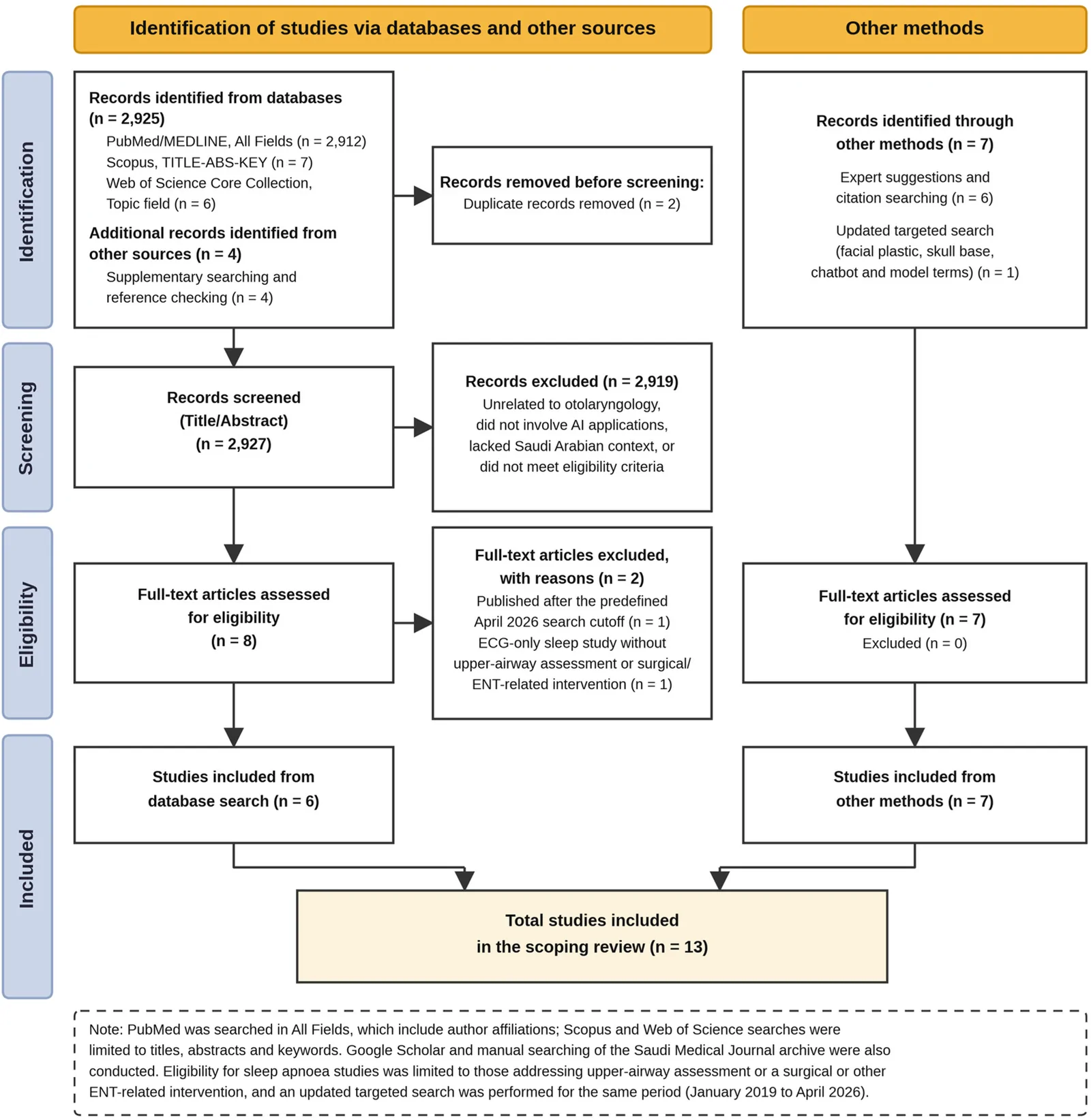
PRISMA flow diagram of study selection.

### Study characteristics

The literature search identified thirteen studies published between 2021 and 2026 that met the inclusion criteria. The studies varied considerably in study design, clinical application, AI methodology, and level of Saudi involvement. The included evidence comprised Saudi cross-sectional studies assessing AI awareness and implementation, Saudi-affiliated AI development studies, evaluations of LLM chatbots for postoperative patient education, and a Saudi-authored systematic review of AI applications in chronic rhinosinusitis. The evidence was distributed across several otolaryngology domains, including cochlear implantation and hearing, rhinology and skull base surgery, facial plastic surgery, laryngology and voice, head and neck oncology, and AI education and implementation. The characteristics of the thirteen included studies are summarised in Table 2.

**Table 2:** Characteristics of included studies.

Author	Year	ENT domain	Country/context	Study design	AI focus	Participants/dataset	Main findings
Alomary et al. (4)	2021	AI education/ENT	Saudi Arabia	Cross-sectional survey	ML awareness and perceptions	538 medical students	General ML awareness was relatively good, but familiarity with ML applications in otolaryngology was more limited; participants generally supported AI education.
AlSharhan et al. (18)	2024	AI implementation	Saudi Arabia	Cross-sectional survey	AI knowledge and attitudes	110 ENT residents, fellows and consultants	Participants demonstrated favourable attitudes towards AI, although formal training and AI knowledge remained limited.
Alshehri et al. (19)	2024	AI-assisted diagnosis	Saudi Arabia	Cross-sectional survey	AI-assisted diagnostic tools	458 ENT physicians, surgeons and audiologists	Positive perceptions towards AI implementation were reported despite concerns regarding barriers and clinical integration.
Alohali et al. (20)	2023	Cochlear implantation	Saudi Arabia	Machine-learning model development	Prediction of postoperative electrode impedance	80 paediatric cochlear implant recipients	ML models demonstrated feasibility for predicting electrode impedance at multiple postoperative time points; Bayesian linear regression and neural networks performed particularly well across channels.
Ksibi et al. (21)	2023	Laryngology and voice	Saudi-affiliated; international dataset	Deep-learning model development	Voice pathology detection	Saarbruecken Voice Database	A two-level CNN-RNN architecture incorporating gender information achieved 88.84% accuracy and 87.39% F1 score.
Alanazi et al. (22)	2026	Rhinology	Saudi-authored secondary evidence	Systematic review	AI-based CT interpretation of CRS	6 studies; 2,178 patients	AI models showed promising diagnostic performance, but all six included studies relied on internal validation only, and none was conducted in Saudi Arabia (five in China, one in the United States).
Olatunji et al. (23)	2021	Head and neck oncology (thyroid cancer)	Saudi Arabia; Saudi patient data	Machine-learning model development	Pre-symptomatic prediction of thyroid cancer	218 thyroid cancer patient records, King Fahad Specialist Hospital, Dammam	Random forest achieved the highest accuracy (90.91%) using seven of 15 features; ANN 88.64%, SVM 84.09%, naïve Bayes 81.82%. Single centre; no external validation.
Albehairi et al. (24)	2025	Facial plastic/otology (otoplasty)	Saudi Arabia	Cross-sectional evaluation of chatbot responses	ChatGPT-4 for postoperative patient questions	Five common post-otoplasty questions; otolaryngologist raters	All responses were rated clear and understandable; accuracy was slightly lower for more complex questions (mean 4.2 vs 4.6).
Alghamdi et al. (25)	2024	Facial plastic (rhinoplasty)	Saudi Arabia	Cross-sectional evaluation of chatbot responses	ChatGPT-4 for postoperative patient questions	Five common post-rhinoplasty questions; three experienced raters	All five responses were rated accurate, clear and relevant; reliability was judged comparable to a senior facial plastic surgeon.
Almousa et al. (26)	2026	Facial plastic (facelift)	Saudi Arabia	Comparative evaluation of chatbot responses	ChatGPT-4 vs Gemini for postoperative patient questions	Five postoperative facelift questions; five facial plastic surgeon raters (yes/no scoring)	Accuracy 88% for both platforms; clarity 80% (ChatGPT-4) vs 92% (Gemini); considered useful adjuncts, not replacements for clinicians.
Alsudays et al. (27)	2026	Rhinology (endoscopic sinus surgery)	Saudi Arabia	Comparative evaluation of chatbot responses	ChatGPT-4 vs Gemini vs DeepSeek for postoperative sinus care	Ten standardised post-FESS questions from three online sources; 30 responses rated by two blinded rhinologists using the validated QAMAI tool	ChatGPT-4 and DeepSeek comparable for accuracy, relevance and usefulness; ChatGPT-4 rated clearest, DeepSeek most complete; Gemini performed least well. DeepSeek responses significantly longer; formal readability scores (Flesch Reading Ease, grade level) did not differ between platforms. Only two raters; single-scenario design.
Alamri et al. (28)	2026	Skull base surgery (endoscopic transnasal)	Saudi Arabia	Comparative evaluation using validated instruments	ChatGPT vs DeepSeek vs Gemini postoperative instructions	Two outputs per platform; FKGL/FKRE and PEMAT-P	Grade level 9.1 to 10.9; understandability 76.5% (ChatGPT, DeepSeek) vs 63.3% (Gemini; *p* = 0.005); actionability ≤58.5%; none met patient-education standards.
Alfallaj et al. (29)	2026	Rhinology/skull base surgery	Saudi Arabia; Saudi patient data	Retrospective model development	BERT-based NLP vs logistic regression for CSF-leak prediction	116 skull base surgery patients, King Saud University Medical City (2015 to 2020)	BERT: AUC 1.00, sensitivity 1.00, specificity 0.98; logistic regression: AUC 0.85, sensitivity 0.21. Single centre; no independent test cohort.

### AI awareness and educational preparedness

Three of the included studies investigated attitudes, knowledge and readiness of clinicians and medical students to integrate AI into clinical practice or medical education (4, 18, 19). Participants showed moderate AI knowledge and expressed positive attitudes towards implementation of AI in otolaryngology settings.

Undergraduate medical students showed reasonable awareness of machine learning and recognised the potential role of AI in healthcare, although familiarity with specific applications within otolaryngology was more limited. Most participants supported the incorporation of AI-related education into undergraduate medical curricula (4). Similar findings were reported among practising otolaryngologists, where respondents acknowledged AI as an emerging component of future clinical practice and strongly supported structured AI education during residency training (18).

AI knowledge was not evenly distributed among practising otolaryngologists: consultants knew more than residents, suggesting that clinical experience may influence AI literacy and that residents in particular would benefit from structured postgraduate AI training.

These findings suggest that educational readiness currently exceeds technical competence. Although clinicians and students were positive towards AI, formal training opportunities remain limited across undergraduate and postgraduate education. Therefore, AI education should focus not only on basic awareness but also on critical appraisal of AI models, interpretation of AI outputs, model limitations, data privacy, and appropriate clinical use.

### Clinician perceptions and readiness for AI implementation

AlSharhan et al. (18) and Alshehri et al. (19) examined clinicians’ perceptions regarding implementation of AI within otolaryngology practice. Both studies reported generally favourable attitudes towards AI. Respondents largely viewed AI as a supportive clinical tool that could improve diagnostic accuracy and clinical decision-making rather than replace physician expertise.

Alshehri et al. (19) found that about 61% of respondents reported familiarity with AI-assisted diagnostic tools. Most clinicians expressed confidence in interpreting AI-assisted recommendations and believed AI could improve diagnostic efficiency and patient management. Similarly, AlSharhan et al. (18) identified a positive association between AI knowledge and clinician attitudes, with consultants demonstrating significantly higher knowledge levels than residents. These findings suggest that familiarity with AI may facilitate clinical acceptance. However, positive attitudes should not be interpreted as evidence of successful implementation. Survey studies measure perceptions and self-reported readiness but do not establish whether AI improves diagnostic accuracy, patient outcomes, workflow efficiency, or healthcare costs.

Despite favourable attitudes, respondents consistently emphasised that AI should function as a decision-support system under clinician supervision rather than as an autonomous diagnostic technology.

### Large language models and AI-assisted patient education

Five studies tested general-purpose large language model (LLM) chatbots as a source of postoperative advice for patients after otoplasty (24), rhinoplasty (25), facelift (26), endoscopic sinus surgery (27) and endoscopic transnasal skull base surgery (28). All came from Saudi otolaryngology teams and all included ChatGPT; the more recent ones also compared it with Google Gemini and DeepSeek. Taken together, patient education is now the largest single group of clinically oriented AI studies in this review.

The two earliest studies followed much the same design. Five common postoperative questions were put to ChatGPT-4, and otolaryngologists rated the answers for accuracy, clarity and relevance. After otoplasty, every answer was judged clear and understandable, although accuracy dipped slightly for the more complex questions (24). After rhinoplasty, all five answers were rated accurate and relevant, and the authors felt that ChatGPT-4 was about as reliable as a senior facial plastic surgeon (25). Almousa et al. (26) compared ChatGPT-4 with Gemini after facelift surgery. Both were 88% accurate and Gemini's answers were rated clearer (92% vs. 80%), although none of the differences between the two platforms were statistically significant. The questions themselves had been generated by ChatGPT, answers were scored only as yes or no, and the study was limited to English. Alsudays et al. (27) put ten standardised questions about care after functional endoscopic sinus surgery (FESS) to ChatGPT-4, Gemini and DeepSeek. ChatGPT-4 and DeepSeek scored similarly for accuracy, relevance and usefulness; ChatGPT-4 was rated clearer and DeepSeek more complete, and Gemini did least well. DeepSeek gave significantly longer answers, but formal readability scores did not differ between the three platforms. This study was stronger in design than the others, because two blinded rhinologists rated the answers with a validated instrument (the Quality Analysis of Medical Artificial Intelligence tool, QAMAI). However, it still relied on only two raters and a single clinical scenario.

Alamri et al. (28) approached the question differently. Rather than relying on clinician ratings, they judged chatbot-written postoperative instructions for endoscopic transnasal skull base surgery against validated tools. All three platforms wrote at a Flesch-Kincaid grade level of 9.1–10.9, well above the sixth-grade level usually recommended for patient materials. Understandability was 76.5% for ChatGPT and DeepSeek but only 63.3% for Gemini, and no platform scored above 58.5% for actionability. None met accepted standards for patient education material, and the authors concluded that a clinician should review such content before it reaches patients. Their more sobering results suggest that the very positive findings of the earlier studies owe something to small question sets and informal rating methods.

### AI in cochlear implantation and hearing

Cochlear implantation provides the clearest Saudi example of clinical AI development. Alohali et al. (20) used machine learning to predict postoperative electrode impedance in 80 paediatric cochlear implant recipients with MED-EL FLEX28 electrode arrays. They compared Bayesian linear regression, linear regression, boosted decision tree regression, decision forest regression and neural networks for predicting impedance at one, three, six and twelve months after surgery. Bayesian linear regression and neural networks performed best, with 83% to 100% of predictions falling within an error range of 0–3 kΩ in half of the channels (20).

These findings suggest that machine learning could support postoperative cochlear implant monitoring, as accurate impedance prediction may help clinicians recognise abnormal impedance patterns early. However, the study was retrospective and based on a small single-centre dataset, a limitation the authors acknowledged.

Outside the included evidence, a recent systematic review reported that AI-based applications may benefit deaf and hard-of-hearing people through hearing aids, cochlear implants, sign-language translation, hearing-loss prediction and academic support (30). Cochlear implantation therefore represents an important foundation for Saudi ENT-AI research. Nonetheless, larger Saudi-specific datasets, multicentre validation and longitudinal integration of audiological and functional outcomes are required before such models can be considered for routine clinical implementation.

### AI in rhinology and skull base surgery

Rhinology represents another emerging area of AI research, particularly in the interpretation of computed tomography (CT) images for chronic rhinosinusitis (CRS). Alanazi et al. (22) conducted a systematic review of AI models used for CT-based CRS diagnosis. Six studies involving 2,178 patients were included. Most studies used convolutional neural networks, ResNet-based models, or hybrid deep-learning approaches. Reported sensitivity ranged from 11.1% to 98.1%, specificity from 86.4% to 98.7%, and accuracy from 63.6% to 98.4%, while AUC values reached 0.99 (22). However, these results should be interpreted with caution because there was substantial methodological heterogeneity, and all six studies relied on internal validation (holdout or cross-validation) within single-centre datasets, with none externally validated. None of the primary studies used Saudi data: five were conducted in China and one in the United States. The review is included here because it was produced by a Saudi otolaryngology team, and it shows that Saudi CT data are so far absent from this literature. Future studies should prioritise locally representative CT datasets, multicentre validation and integration of imaging with clinical information.

Moving beyond secondary evidence, Alfallaj et al. (29) built a BERT-based natural language processing model to predict cerebrospinal fluid (CSF) leak after endoscopic endonasal skull base surgery. They used records from 116 patients treated at King Saud University Medical City between 2015 and 2020. The model comfortably outperformed conventional logistic regression (AUC 1.00 vs. 0.85; sensitivity 1.00 vs. 0.21; specificity 0.98 vs. 0.91), and it is the only study in this review to build a rhinology or skull base model from Saudi surgical patients. That said, a perfect AUC in a small single-centre dataset, tested with cross-validation and no independent test cohort, raises the possibility of overfitting, and the authors themselves call for external validation before clinical use.

### AI in laryngology and voice

Ksibi et al. (21) developed a deep-learning model for automated voice pathology detection using a two-level CNN-RNN architecture. The study was conducted by researchers affiliated with Princess Nourah bint Abdulrahman University, Jazan University, King Khalid University and King Saud University. The model used the Saarbruecken Voice Database and incorporated gender information into a two-level classification system. The first level classified the speaker according to gender, while the second classified the voice as healthy or pathological. The proposed model achieved an accuracy of 88.84% and an F1 score of 87.39% (21). No otolaryngologists were involved and the recordings came from a German database; the study was included because it addresses a laryngological condition and was conducted at Saudi institutions. Its performance on Arabic-speaking Saudi patients therefore remains untested.

### AI in head and neck oncology

Head and neck oncology is still thinly covered in the Saudi AI literature, and we found only one primary study. Olatunji et al. (23) used the records of 218 patients from King Fahad Specialist Hospital in Dammam to build machine-learning models that predict thyroid cancer before symptoms appear. Random forest performed best, reaching 90.91% accuracy with seven of the 15 available features, followed by an artificial neural network (88.64%), a support vector machine (84.09%) and naïve Bayes (81.82%). The work came from a computer-science team, relied on a small single-centre dataset and was not externally validated. We found no Saudi study applying AI to the detection, imaging, staging or surgical management of mucosal head and neck cancer, even though these uses are well established internationally, from tumour detection and segmentation to treatment planning and outcome prediction. This remains one of the clearest opportunities for Saudi ENT-AI research.

### AI in surgical and ENT-related management of obstructive sleep apnoea

No eligible study used AI in the otolaryngological assessment or management of obstructive sleep apnoea (OSA), for example in upper-airway evaluation, drug-induced sleep endoscopy or surgical planning. The Saudi-affiliated OSA studies we came across during screening came from sleep medicine and engineering. They focused on detecting apnoeic events from signals such as the electrocardiogram, with no upper-airway assessment or ENT input, and so fell outside the scope of this review. Given how central otolaryngologists are to the evaluation and surgical treatment of OSA, AI tools for upper-airway phenotyping, endoscopic assessment and predicting surgical outcomes are an obvious gap for Saudi ENT research to fill.

### Barriers to AI adoption

Three studies identified common barriers limiting wider implementation of AI in Saudi otolaryngology (4, 18, 19). Educational barriers represented the most frequently reported challenge, including insufficient AI training, lack of curriculum integration, and limited familiarity with AI technologies. Implementation-related barriers included concerns regarding diagnostic reliability, interoperability with existing hospital systems, financial costs, data privacy, and technical support. Respondents also highlighted uncertainty regarding responsibility for AI-assisted clinical decisions and emphasised the importance of maintaining physician oversight during AI-supported diagnosis.

Table 3 presents an evidence map illustrating the thematic distribution of the included studies across the four predefined thematic domains and the added patient-education domain. Clinical AI development and LLM-based patient education are the largest domains, with five studies each, followed by AI awareness and educational preparedness and by barriers to adoption, with three each. However, only four of the clinical AI development sources are primary model-development studies, and all of them used small single-centre or public datasets.

**Table 3:** Evidence map of artificial intelligence research in Saudi otolaryngology.

Thematic domain	n	Included studies	Summary of evidence and gaps
AI awareness and educational preparedness	3	Alomary et al. (4); AlSharhan et al. (18); Alshehri et al. (19)	Medical students and otolaryngologists were aware of AI and supported AI teaching, but formal training was limited; consultants were more knowledgeable than residents.
Clinician perceptions and readiness for AI implementation	2	AlSharhan et al. (18); Alshehri et al. (19)	Attitudes were favourable and AI was seen mainly as decision support; about 61% of respondents reported familiarity with AI-assisted diagnostic tools. Perceptions do not show clinical benefit.
Clinical AI development	5	Olatunji et al. (23); Alohali et al. (20); Ksibi et al. (21); Alfallaj et al. (29); Alanazi et al. (22)	Models for thyroid cancer prediction, cochlear implant impedance, voice pathology and CSF-leak prediction, plus a systematic review of CT-based CRS diagnosis. High reported accuracy, but small single-centre or public datasets and no external validation.
AI-assisted patient education (LLMs)	5	Alghamdi et al. (25); Albehairi et al. (24); Almousa et al. (26); Alsudays et al. (27); Alamri et al. (28)	ChatGPT, Gemini and DeepSeek generally gave accurate postoperative advice after otoplasty, rhinoplasty, facelift, sinus and skull base surgery. Small question sets and mostly unvalidated rating scales; readability and actionability fell short of standards when validated tools were used; no Arabic testing.
Barriers to AI adoption	3	Alomary et al. (4); AlSharhan et al. (18); Alshehri et al. (19)	Limited training, concerns about reliability, integration with hospital systems, cost, data privacy and medico-legal responsibility.

Studies may contribute to more than one domain; 13 unique studies were included.

CRS, chronic rhinosinusitis; CSF, cerebrospinal fluid; CT, computed tomography; LLM, large language model.

## Discussion

### Principal findings

To our knowledge, this is the first scoping review to map AI research in Saudi otolaryngology. Thirteen studies met the eligibility criteria, which shows that the field is still small. Three patterns stand out. First, the survey studies show that clinicians and students are positive about AI but report little formal training. Second, the fastest-growing group consists of studies testing LLM chatbots as a source of postoperative information for patients. Third, only a handful of studies have developed AI models, mostly on small single-centre or public datasets, and none has been externally validated or tested prospectively. Head and neck oncology and the ENT management of obstructive sleep apnoea remain largely untouched.

### Comparison with the wider literature

The gap between enthusiasm and training is not unique to Saudi Arabia. Saudi otolaryngologists and medical students supported AI and wanted it taught, yet few had received formal training (4, 18, 19). Similar findings have been reported among Saudi health professionals more broadly (31) and in otolaryngology training internationally, which led the AAO-HNS Artificial Intelligence Task Force to describe AI literacy as a core competency for modern ENT practice (32, 33). Positive attitudes are a good starting point, but they are not evidence that AI improves care.

The model-development studies reflect the implementation gap seen across otolaryngology, where technical performance has run ahead of clinical evidence (34). International reviews have found that most otolaryngology AI studies are retrospective, single-centre and internally validated (35, 36), and the Saudi studies are no exception. The cochlear implant, voice, chronic rhinosinusitis, skull base and thyroid models identified here (20–23, 29) are best read as proofs of concept. The chatbot studies raise a different problem. All asked ten or fewer questions and most relied on small panels of clinician raters without a validated instrument; their near-perfect scores contrast with the weaker results obtained when validated readability and understandability tools were used (28). A study published shortly after our search period also found poor agreement between ChatGPT-4o and a radiologist in reading sinus CT scans (37). None of the chatbot studies tested Arabic, which matters because many Saudi patients are likely to use these tools in Arabic.

### Implications for implementation and governance

Several practical steps follow. AI tools should enter ENT practice as decision support under clinician oversight, and only after external validation in Saudi patients. Chatbot-generated patient information should be reviewed by clinicians before it is used, and departments may prefer to provide vetted, bilingual postoperative material rather than leave patients to consult chatbots on their own. At the system level, AI software that informs diagnosis or treatment falls under the Saudi Food and Drug Authority's guidance on AI- and machine-learning-based medical devices (38), and its use should follow the national AI ethics principles issued by SDAIA (39). Hospitals will also need clear local policies on data privacy, accountability for AI-assisted decisions and ongoing monitoring of model performance, which were among the main concerns raised by clinicians in this review and in the wider literature (40, 41). Finally, structured AI teaching, covering how models work, their limitations and how to appraise them, should be built into undergraduate and residency curricula.

### Future directions and collaboration

Many of these gaps are too large for any one department to close. Saudi academic ENT departments could pool de-identified imaging, endoscopic, audiological and surgical data into a shared multicentre resource, so that a model developed at one centre can be tested at others. Privacy-preserving approaches such as federated learning would make this possible without moving patient records between institutions. A national ENT-AI research network, ideally working with the national otolaryngology society, could agree common data standards, coordinate external validation studies, and develop and test Arabic-language patient information. Closer partnerships with computer-science and engineering departments, which already produce much of the Saudi-affiliated technical work, would help ensure that future models answer clinically important questions. Priority areas include head and neck oncology, upper-airway assessment in obstructive sleep apnoea, and prospective studies that measure patient outcomes rather than accuracy alone.

### Strengths and limitations

This review has several strengths. To our knowledge, it is the first to map AI research in Saudi otolaryngology, it followed the Arksey and O'Malley framework with PRISMA-ScR reporting, and it covered clinical, educational and patient-education research. It also has limitations. The number of eligible studies was small, and most were cross-sectional surveys or chatbot evaluations rather than studies of clinical outcomes. The AI development studies relied on small single-centre or publicly available datasets, and none was externally validated. Our original search terms did not capture facial plastic and skull base applications or specific chatbot names; a supplementary search addressed this, but relevant studies, particularly in engineering and computer-science journals, may still have been missed. Because the Scopus and Web of Science searches were limited to titles, abstracts and keywords, studies that mention Saudi Arabia only in author affiliations may have been missed in those databases, although the PubMed All Fields search and the author-affiliation search partly offset this. We included only English-language publications because English is the primary language of medical education, practice and research publication in Saudi Arabia; nevertheless, a small number of reports in other languages may have been missed. The available evidence is therefore not yet sufficient to judge the real-world effectiveness of AI in Saudi otolaryngology.

## Conclusion

AI research in Saudi otolaryngology is growing but remains at an early stage. Clinicians and students are positive about AI, and Saudi teams have begun to study LLM-based patient education and AI models in cochlear implantation, rhinology and skull base surgery, laryngology and head and neck oncology. These studies, however, rely on small and often non-Saudi or public datasets, lack external validation and have not been evaluated prospectively, and none has yet addressed surgical or other ENT-related interventions for obstructive sleep apnoea.

Moving AI from promising technology into routine practice will require structured AI education, Saudi-specific datasets, prospective multicentre studies with external validation, rigorous bilingual (Arabic and English) evaluation of LLM-generated patient information, and clear national governance. Head and neck oncology and the ENT management of obstructive sleep apnoea deserve particular attention. Such efforts would support Saudi Vision 2030 by promoting the safe, evidence-based and patient-centred use of AI in otolaryngology.
